# The association between blood pressure, renal function, and subclinical atherosclerosis in young and middle-aged non-hypertensive individuals: a mediation analysis

**DOI:** 10.3389/fcvm.2025.1613785

**Published:** 2025-10-20

**Authors:** Wentao Lu, Wen Li, Zhuoqun Zou, Qinyuan Yang, Jianguang Tian

**Affiliations:** ^1^ Rehabilitation Department, Shanghai Health and Medical Center, Wuxi, Jiangsu, China; ^2^ Physical Diagnosis Department, Shanghai Health and Medical Center, Wuxi, Jiangsu, China; ^3^ Gerontology Department, Shanghai Health and Medical Center, Wuxi, Jiangsu, China; ^4^ Section of Science and Education, Shanghai Health and Medical Center, Wuxi, Jiangsu, China

**Keywords:** blood pressure, renal function, subclinical atherosclerosis, mediation analysis, non-hypertensive individuals

## Abstract

**Background:**

This study aimed to investigate the interactions between blood pressure, renal function, and subclinical atherosclerosis in young and middle-aged individuals without hypertension.

**Methods:**

A total of 1,590 young and middle-aged individuals without hypertension were selected from the Shanghai Health and Medical Center database. Univariate and multivariate regression analyses were used to identify risk factors associated with subclinical atherosclerosis, and a mediation analysis using the bootstrap method was conducted to assess the mediating role of renal function between blood pressure and subclinical atherosclerosis.

**Results:**

The multivariate linear regression analysis identified significant correlations with age, gender, diastolic blood pressure (DBP), systolic blood pressure (SBP), glomerular filtration rate (GFR), history of cardiovascular disease (CVD), glycated hemoglobin (HbA1c), smoking history, and brachial-ankle pulse wave velocity (baPWV) (*P* < 0.05). The results indicated that GFR mediated the relationships of both systolic blood pressure (SBP) and diastolic blood pressure (DBP) with brachial-ankle pulse wave velocity (baPWV) (*P* < 0.05). The same is true among males after stratification by gender.

**Conclusion:**

Renal function is independently associated with blood pressure and subclinical atherosclerosis after adjusting for confounders. Moreover, renal function mediates the relationship between blood pressure and subclinical atherosclerosis, also in males.

## 1 Introduction

Due to lifestyle changes, abnormal blood pressure in young and middle-aged, non-hypertensive populations is receiving increasing attention. Although this group is often considered low-risk, studies suggest that even when blood pressure does not meet the clinical diagnostic criteria for hypertension, fluctuations and abnormalities in blood pressure may still pose a potential threat to cardiovascular health (1). Previous research has demonstrated a link between decreased renal function and the development of atherosclerosis, with dysarteriotony potentially accelerating this process (2). Therefore, abnormal blood pressure and declining renal function are interconnected factors contributing to the progression of subclinical atherosclerosis. Subclinical atherosclerosis represents an early change related to atherosclerotic cardiovascular disease (ASCVD), and identifying it early, along with timely intervention, can help delay serious vascular complications (3). However, few studies have examined how blood pressure, renal function, and atherosclerosis interact in young- and middle-aged individuals without hypertension; thus, it remains unclear whether blood pressure directly affects GFR and influences cardiovascular health through reduced renal function.

This study aims to explore the interactions between blood pressure, renal function, and subclinical atherosclerosis in this population and clarify the role of renal function. The findings will enhance our understanding of cardiovascular risk in non-hypertensive individuals and provide a theoretical basis for early intervention.

## 2 Materials and methods

### 2.1 Study population

This study selected 1,590 young and middle-aged individuals aged 20–59 years who did not have hypertension and participated in health checkups at the Shanghai Health and Medical Center between January 2017 and December 2023. These samples mainly include workers in the Shanghai region. Inclusion criteria included: (1) Complete health checkup data from 2017; (2) Initial blood pressure measurements showing systolic blood pressure (SBP) less than 140 mmHg and diastolic blood pressure (DBP) less than 90 mmHg, without the use of antihypertensive medication. The exclusion criteria were: (1) Age at initial assessment of 60 years or older, or under 20 years; (2) Abnormal ankle-brachial index (ABI less than 0.9 or greater than 1.4) or confirmed peripheral arterial disease; (3) White coat hypertension (elevated office blood pressure but normal out-of-office readings); (4) Significant discrepancies between office blood pressure and brachial-ankle pulse wave velocity (baPWV) measurements, defined as differences of 20 mmHg systolic or 10 mmHg diastolic or more; (5) Unexplained abnormal baPWV values; (6) Episodes of acute illness or exacerbations of chronic diseases; (7) Severe heart failure, arrhythmia, coronary artery disease, diabetes, stroke, or renal insufficiency. The study protocol received approval from the ethics committee of the Shanghai Health and Medical Center.

### 2.2 Recording of cardiovascular risk factors

Clinical data collection for this study was based on health checkup records and questionnaire data. The Shanghai Health and Medical Center provided health checkup records including participants' basic information, physical examination results, and medical history. Additionally, questionnaires collected self-reported data on lifestyle, smoking status, and other cardiovascular risk factors. The health checkup records and questionnaire data were linked for each participant and cross-validated using standardized procedures, ensuring that the data were comprehensive in scope and accurate in detail.

### 2.3 Blood pressure measurement and recording methods

This study employed standardized methods for measuring blood pressure in an office setting. Participants sat with their arms relaxed. Measurements were taken after at least 5 min of rest in a quiet environment. Calibrated electronic blood pressure monitors, such as the Omron HEM-907 model, were used. Additionally, the room temperature was controlled to prevent external interference. Systolic blood pressure (SBP) and diastolic blood pressure (DBP) were measured twice for each participant. The average of these measurements was calculated to enhance accuracy and reliability. All blood pressure values were recorded in millimeters of mercury (mmHg). If the difference between the two measurements exceeded 20 mmHg for systolic or 10 mmHg for diastolic blood pressure, a third measurement was taken for verification. All measurements were conducted by trained medical personnel to ensure consistency and standardization. The results were recorded in electronic health files for further analysis.

### 2.4 Biochemical indicators test reagents

The biochemical indicators used in this study include aspartate aminotransferase (AST) and alanine aminotransferase (ALT), commonly used to assess liver function. We measured triglycerides (TG), an important blood lipid indicator, because changes in triglyceride concentration are closely linked to cardiovascular disease risk. We used glycated hemoglobin (HbA1c) to assess blood glucose control in diabetic patients, as it reflects the average blood glucose level over the past few months. Total triiodothyronine (T3) was measured to evaluate thyroid function, with abnormal levels potentially associated with various metabolic diseases. Creatinine (Cr), a common indicator of renal function, was measured to assess glomerular filtration. All biochemical indicators were measured using fasting blood samples collected through venipuncture to ensure accurate and reliable results. Based on serum creatinine, the estimated glomerular filtration rate (GFR) was then calculated using the CKD-EPI equation (4).

### 2.5 Arterial stiffness detection

Brachial-Ankle Pulse Wave Velocity (baPWV) is a key indicator for assessing arterial stiffness. It reflects the condition of the arteries by measuring the speed of the pulse wave traveling from the brachial artery to the ankle artery. Arterial stiffness was measured using the Omron BP-203RPE II VP-1000 automatic oscillometric device for baPWV measurement. Measurements were conducted in a quiet environment to ensure accurate and reliable results. Oscillometric cuffs were applied to both upper arms and ankles, and blood pressure changes were simultaneously recorded to calculate the baPWV value. We followed the reference standard outlined in the American College of Cardiology's 1993 report (5). A baPWV of ≥1,400 cm/s indicates abnormally high arterial stiffness.

### 2.6 Statistical methods

In this study, quantitative variables that follow a normal distribution are represented by the mean ± standard deviation, while those that do not follow a normal distribution are represented by the median and interquartile range [M (P25, P75)]. Qualitative variables are expressed as absolute frequencies and percentages. In the data analysis process, we performed descriptive statistical analysis using SPSS software to calculate the mean, standard deviation, and correlation coefficients for each variable. To further analyze the data, we used the BruceR package in R. First, we examined the effects of independent variables on dependent and mediating variables separately. Then, we performed significance tests for the mediating effects. We used the bootstrap method, which employs repeated sampling to calculate confidence intervals for mediation effects, enabling us to determine whether the mediation effect is significant. All statistical analyses employed two-tailed tests, with the significance level set at 0.05 (α = 0.05).

## 3 Results

### 3.1 Baseline characteristics

A total of 1,590 individuals were included in this study. Among them, 1,070 were male, accounting for 67.3% of the participants. The median age was 48 years, with an interquartile range of 42–53 years. There were 421 smokers, accounting for 26.5% of participants, and 284 drinkers, accounting for 17.9% of participants.

### 3.2 Risk factors for subclinical atherosclerosis

In Table 1, the indicators for individuals with subclinical atherosclerosis are compared to those for individuals without it. A *P*-value less than 0.05 in the table indicates a statistically significant association between the indicator and subclinical atherosclerosis. As shown in Table 1, the following indicators were associated with subclinical atherosclerosis: systolic blood pressure, diastolic blood pressure, heart rate, glomerular filtration rate, waist-to-hip ratio, age, sex, uric acid to creatinine ratio, AST to ALT ratio, triglycerides, total cholesterol, low-density lipoprotein, high-density lipoprotein, glycated hemoglobin, family history of cardiovascular disease, smoking history, and alcohol consumption.

**Table 1:** Comparison of indicators under subclinical atherosclerosis grouping.

Indicators	No atherosclerosis (*n* = 1,177)	Atherosclerosis (*n* = 413)	*t/Z*	*P*
Systolic Blood Pressure (SBP)	115.11 ± 11.17	124.54 ± 9.43	−16.6261	<0.001
Diastolic Blood Pressure (DBP)	69.93 ± 8.07	75.78 ± 7.79	−12.797	<0.001
Glomerular Filtration Rate (GFR)	105.41 ± 22.29	101.07 ± 19.78	3.499	<0.001
Waist-to-Hip Ratio	0.86 ± 0.06	0.89 ± 0.06	−8.126	<0.001
Age	45.70 ± 7.74	49.86 ± 6.70	−10.413	<0.001
Uric Acid/Creatinine Ratio	4.48 ± 1.56	4.17 ± 1.33	3.907	<0.001
Body Mass Index (BMI)	24.06 ± 2.99	24.27 ± 2.75	−1.240	0.215
Heart Rate	74.46 ± 10.11	78.52 ± 10.92	−6.856	<0.001
Aspartate Aminotransferase (AST)	21.51 ± 6.96	23.10 ± 7.16	−3.969	<0.001
Alanine Aminotransferase (ALT)	22.73 ± 15.10	26.29 ± 16.19	−4.039	<0.001
AST/ALT Ratio	1.14 ± 0.46	1.05 ± 0.42	3.862	<0.001
Triglycerides (TG)	1.42 ± 1.18	1.73 ± 1.25	−4.406	<0.001
Total Cholesterol	4.92 ± 0.90	5.05 ± 0.93	−2.477	0.013
Low-Density Lipoprotein (LDL)	2.91 ± 0.63	3.02 ± 0.67	−2.965	0.003
High-Density Lipoprotein (HDL)	1.40 ± 0.33	1.35 ± 0.34	2.616	0.009
Glycated Hemoglobin (HbA1c)	5.58 ± 0.27	5.67 ± 0.30	−5.065	<0.001
Urea Nitrogen	5.31 ± 1.11	5.40 ± 1.08	−1.459	0.145
Uric Acid	312.41 ± 86.22	304.11 ± 82.68	1.701	0.089
Thyroid-Stimulating Hormone (TSH)	2.19 ± 1.50	2.15 ± 1.13	0.467	0.640
Serum cystatin	0.79 ± 0.11	0.81 ± 0.10	−0.608	0.544
Homocysteine	9.05 ± 4.70	8.78 ± 3.51	0.649	0.517
Gender (Male, %)	746 (63.4)	324 (78.5)	31.543	<0.001
Family History of CVD, *n* (%)	679 (57.7)	271 (65.6)	7.911	0.005
Smoking History, *n* (%)	270 (22.9)	151 (36.6)	29.142	<0.001
Alcohol Consumption History, *n* (%)	191 (16.2)	93 (22.5)	8.246	0.004
Diet Habits, *n* (%)				
Mixed Diet	1,040 (88.4)	357 (86.4)		
Predominantly Vegetarian	80 (6.8)	33 (8.0)	1.061	0.588
Predominantly Meat-based	57 (4.8)	23 (5.6)		
Regular Exercise, *n* (%)	684 (58.1)	253 (61.3)	1.250	0.264
ASCVD Risk Level				
Low Risk = 1	1,142 (97.0)	403 (97.6)		
Moderate Risk = 2	24 (2.0)	7 (1.7)	−0.583	0.560
High Risk = 3	11(0.9)	3(0.7)		

### 3.3 Multivariate regression analysis with baPWV as the dependent variable

Table 2 shows the results of a multivariate linear regression analysis with baPWV as the dependent variable: age, gender, SBP, DBP, GFR, family history of cardiovascular disease (CVD), HbA1c, and smoking history were significantly correlated with baPWV (*P* < 0.05).

**Table 2:** Multivariate linear regression analysis with baPWV as the dependent variable.

Independent variable	Unstandardized coefficients		Standardized coefficients	*t*	*P*
	B	Standard error	Beta		
SBP	4.535	0.538	0.281	8.425	0.000
Age	7.031	0.706	0.271	9.960	0.000
DBP	4.634	0.728	0.206	6.366	0.000
Gender	27.620	10.517	0.072	2.626	0.009
GFR	−0.772	0.231	−0.089	−3.347	0.001
Family History of CVD (Yes = 1)	23.198	8.952	0.060	2.591	0.010
HbA1c	36.814	16.078	0.056	2.290	0.022
Smoking History (Yes = 1)	23.847	11.070	0.056	2.154	0.031

### 3.4 Pearson correlation analysis

**Figure 1:**
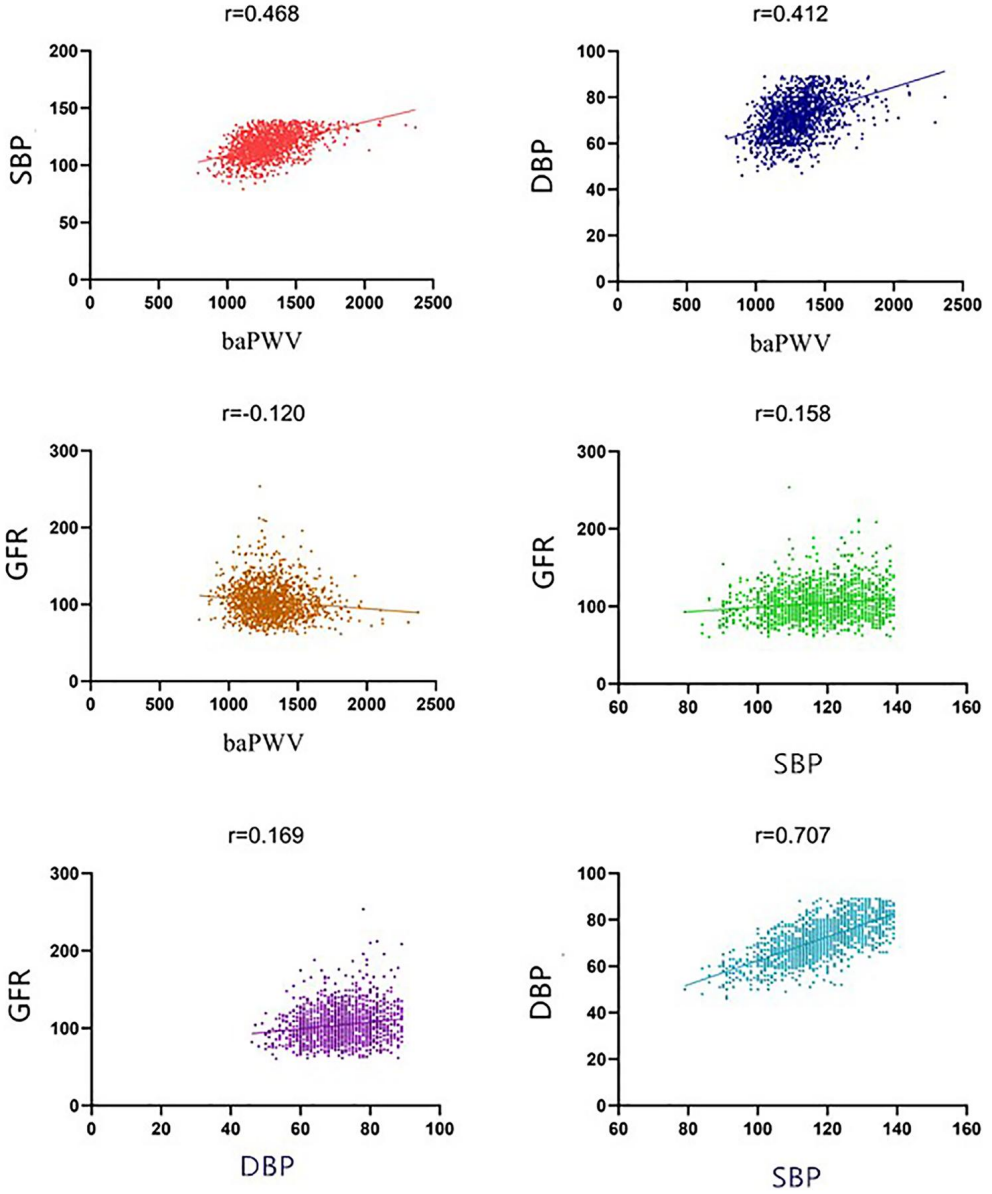
Scatter plot of the relationship between blood pressure, GFR, and baPWV.

Pearson correlation analysis revealed that brachial-ankle pulse wave velocity (baPWV) was positively correlated with systolic blood pressure (SBP) and diastolic blood pressure (DBP), with correlation coefficients of 0.468 and 0.412, respectively (*P* < 0.001). Additionally, baPWV demonstrated a negative correlation with the Glomerular Filtration Rate (GFR), with a correlation coefficient *r* = −0.120 (*P* < 0.001), as shown in Table 3. Furthermore, GFR was positively correlated with SBP and DBP, with correlation coefficients of 0.158 and 0.169, respectively (*P* < 0.001), as shown in Table 4. Figure 1 presents scatter plots that illustrate the relationships among blood pressure, GFR, and baPWV, visually illustrating the significant correlations identified.

**Table 3:** The correlation between baPWV and blood pressure and renal function indicators.

Indicators	Correlation coefficient (*r*)	*P*
SBP	0.468	<0.001
DBP	0.412	<0.001
GFR	−0.120	<0.001

**Table 4:** The correlation between GFR and blood pressure.

Indicators	Correlation coefficient (*r*)	*P*
SBP	0.158	<0.001
DBP	0.169	<0.001

### 3.5 Mediation analysis

We conducted a mediation analysis with baPWV as the dependent variable, SBP as the independent variable, and GFR as the mediating variable, and included age, gender, family history of CVD, smoking history, and HbA1c as covariates. Specifically, Table 5 presents the mediation analysis results. GFR significantly mediated the relationship between SBP and subclinical atherosclerosis (*P* < 0.05). The mediation effect (ab, representing the indirect effect) and the direct effect (c′) had opposite signs. This indicates that although elevated SBP increased baPWV, the positive effect of SBP on baPWV was reduced by an increase in GFR.

**Table 5:** Analysis of the mediating effect of SBP on GFR during subclinical atherosclerosis.

Effect Type	Effect estimate	Standard error	*z*	*P*	*95% CI (Bootstrap)*
Mediation Effect (ab)	−0.160	0.071	−2.267	0.023	(−0.323, −0.043)
Direct Effect (c′)	6.857	0.430	15.947	<0.001	(6.048, 7.739)
Total Effect (c)	6.697	0.416	16.085	<0.001	(5.924, 7.564)

Mediation path: SB*P* → GFR(Mediation variable) → baPWV. Estimate the CI of the effect size using the Bootstrap method with 1,000 iterations and a seed number of 1.

Similarly, when baPWV was treated as the dependent variable and DBP as the independent variable, GFR again showed a significant mediating effect (*P* = 0.015), as illustrated in Table 6.

**Table 6:** Analysis of the mediating effect of DBP on GFR during subclinical atherosclerosis.

Effect Type	Effect estimate	Standard Error	*z*	*P*	*95% CI (Bootstrap)*
Mediation Effect (ab)	−0.242	0.099	−2.439	0.015	(−0.459, −0.066)
Direct Effect (c')	8.971	0.581	15.123	<0.001	(7.647, 9.890)
Total Effect (c)	8.550	0.565	15.130	<0.001	(7.469, 9.670)

Mediation path: DBP → GFR(Mediation variable) → baPWV. Estimate the CI of the effect size using the Bootstrap method with 1,000 iterations and a seed number of 1.

To explore the mediating effect analysis of GFR in different genders, we further stratified it by gender. Among men, we conducted a mediation analysis with baPWV as the dependent variable, SBP as the independent variable, and GFR as the mediating variable, and included age, family history of CVD, smoking history, and HbA1c as covariates. GFR significantly mediated the relationship between SBP and subclinical atherosclerosis in male individuals (*P* < 0.05). Similarly, GFR again showed a significant mediating effect between DBP and subclinical atherosclerosis in male individuals (*P* < 0.05) in Table 7.

**Table 7:** Male: analysis of the mediating effect of BP on GFR during subclinical atherosclerosis.

Effect type	SBP					DBP				
	Effect estimate	Standard error	*z*	*P*	*95% CI (Bootstrap)*	Effect estimate	Standard error	*z*	*P*	*95% CI (Bootstrap)*
Mediation Effect (ab)	−0.285	0.110	−2.606	0.009	(−0.524, −0.099)	−0.319	0.141	−2.270	0.023	(−0.622, −0.083)
Direct Effect (c')	6.691	0.590	11.332	<0.001	(5.617,7.835)	8.024	0.733	10.949	<0.001	(6.653, 9.501)
Total Effect (c)	6.406	0.574	11.160	<0.001	(5.330,7.548)	7.705	0.712	10.817	<0.001	(6.344,9.137)

Mediation path: SBP/DBP → GFR(Mediation variable) → baPWV. Estimate the CI of the effect size using the Bootstrap method with 1,000 iterations and a seed number of 1.

Among women, we conducted a mediation analysis with baPWV as the dependent variable, SBP/DBP as the independent variable, GFR as the mediating variable, and included age, family history of CVD, and HbA1c as covariates. No women had a history of smoking, so the smoking history was excluded from the covariate. Table 8 presents the mediation analysis results. GFR does not mediate the relationship between SBP/DBP and subclinical atherosclerosis in female individuals (*P* > 0.05).

**Table 8:** Female: analysis of the mediating effect of BP on GFR during subclinical atherosclerosis.

Effect type	SBP					DBP				
	Effect estimate	Standard error	*z*	*P*	*95% CI (Bootstrap)*	Effect estimate	Standard error	*z*	*P*	*95% CI (Bootstrap)*
Mediation Effect (ab)	−0.035	0.065	−0.539	0.590	(5.760,8.421)	−0.137	0.119	−1.147	0.251	(−0.411,0.050)
Direct Effect (c')	6.987	0.689	10.146	<0.001	(5.760,8.421)	9.765	0.941	10.377	<0.001	(8.052,11.754)
Total Effect (c)	6.952	0.680	10.227	<0.001	(5.653,8.341)	9.628	0.925	10.424	<0.001	(7.938,11.492)

Mediation path: SBP/DBP → GFR(Mediation variable) → baPWV. Estimate the CI of the effect size using the Bootstrap method with 1,000 iterations and a seed number of 1.

Sensitivity analyses were conducted on all the above mediation models. The confidence interval of ACME included 0, indicating that other potential confounding factors were not sensitive to our terminal models.

## 4 Discussion

Various factors, including lifestyle, genetics, and the environment, influence blood pressure characteristics in young and middle-aged non-hypertensive individuals. Research shows that dietary habits, particularly high salt intake, are linked to elevated blood pressure (6). Additionally, a lack of exercise and obesity significantly contribute to high blood pressure, especially in younger adults (7). Weight gain often leads to significant increases in blood pressure. In modern society, the rapid pace of life and psychological factors such as stress and anxiety further affect blood pressure in this population. Studies have found a strong association between increased psychological stress, anxiety levels, and elevated blood pressure (8). For example, individuals who are chronically stressed may experience overactivity of the sympathetic nervous system, resulting in elevated blood pressure (9). Therefore, maintaining a healthy lifestyle and focusing on mental health are crucial for managing blood pressure in young and middle-aged adults (10).

In addition, genetic factors play an important role in the blood pressure characteristics of young and middle-aged populations (11). Studies indicate a strong correlation between family history and blood pressure levels, especially among younger individuals. Genomic studies suggest that specific genetic variations, such as single nucleotide polymorphisms affecting sodium regulation and vascular tone, are involved in regulating blood pressure. These findings provide new insights into the mechanisms underlying blood pressure variability. Therefore, future research should explore the interaction between genetic and environmental factors, to gain a more comprehensive understanding of blood pressure regulation and its changes in young and middle-aged non-hypertensive individuals.

In this study, brachial-ankle pulse wave velocity (baPWV) is used as a key indicator for assessing arterial stiffness, and its effectiveness has been confirmed by multiple studies. The ESC 2023 (12) guidelines indicate that for every increase of 100 cm/s in baPWV, the risk of cardiovascular events increases by 15%. Research by Keishi et al. (13) found that among 320 patients without hypertension, those in the baPWV ≥ 1,800 cm/s group had a 2.47 times higher risk of having a coronary artery calcification (CAC) score ≥400 compared to the low baPWV group (*P* = 0.047). After a 575-day follow-up, the incidence of cardiovascular events significantly increased in the high baPWV group compared to the low baPWV group. Taken together, these results suggest that elevated baPWV is directly related to the severity of atherosclerosis and the risk of clinical events, further proving the reliability of using baPWV as a standard for assessing arterial stiffness.

In the univariate and multivariate regression analyses for subclinical atherosclerosis, a decline in glomerular filtration rate (GFR) was identified as a significant factor associated with the progression of atherosclerosis. Moreover, research shows that patients with reduced kidney function exhibit greater arterial stiffness compared to those with normal function, underscoring the kidney's role in blood pressure regulation and vascular endothelial function (14). This study offers new insights into how decreased GFR contributes to vascular changes underlying atherosclerosis development.

Research indicates that impaired renal function is closely linked to higher levels of oxidative stress in the body (15). This increase in oxidative stress may accelerate the progression of atherosclerosis (16). Studies show a strong correlation between declining renal function and an increased risk of cardiovascular events, especially in people with hypertension. Since atherosclerosis is a major contributor to cardiovascular events, therefore, improving renal function may help lower the risk of atherosclerosis.

Our study found no significant correlation between body mass index (BMI) and baPWV (*P* > 0.05), while waist-to-hip ratio (WHR) showed a significant correlation (*P* < 0.05). Although BMI is commonly used to assess obesity (17), its effect on atherosclerosis was less significant than that of the WHR in this study. This may be because some individuals with high BMI are physically active and have greater muscle mass, which can lower the risk of atherosclerosis. Consequently, the levels of physical activity in individuals with high BMI may have influenced the correlation findings in this study.

This study investigates the connections between renal function, blood pressure, and subclinical atherosclerosis, as illustrated in Figure 1. The results show that impaired renal function is linked to higher blood pressure and the development of subclinical atherosclerosis. Specifically, individuals with renal impairment often have elevated blood pressure, likely due to the kidneys' reduced ability to regulate fluid and electrolyte balance. Moreover, prior research has established a significant link between impaired renal function and the development of atherosclerosis, underscoring the kidneys' critical role in cardiovascular health (18). Thus, monitoring changes in renal function is essential for the early detection of hypertension and the risk of atherosclerosis.

This study found that renal function plays a significant role in mediating the relationship between blood pressure and atherosclerosis. Specifically, decreased renal function is closely linked to elevated blood pressure, which further promotes the progression of atherosclerosis. This indicates that decreased renal function accelerates atherosclerosis by affecting blood pressure regulation (19, 20). Additionally, decreased renal function can influence the body's endocrine environment, such as through the activation of the renin-angiotensin system, which contributes to increased blood pressure and the progression of atherosclerosis (21).

Our study provides novel mechanistic insights into the complex relationship between blood pressure, kidney function, and subclinical atherosclerosis (measured by baPWV), revealing a significant gender disparity. We found that GFR acts as an important mediator between both SBP and DBP and baPWV, exclusively in male individuals. This suggests that the detrimental effect of elevated blood pressure on arterial stiffness in men may be partially exerted through its impact on renal function. In contrast, GFR did not demonstrate a significant mediating role in the relationship between SBP/DBP and baPWV among women. This critical sex difference aligns with and potentially helps explain the broader epidemiological patterns observed in large-scale studies. The recent comprehensive meta-analysis encompassing 167 studies and 509,743 participants globally confirmed that males exhibit significantly higher levels of both baPWV (+0.77 m/s) and cfPWV (+0.35 m/s) compared to females (22). The reliance on GFR-mediated pathways in men could contribute to their generally higher baseline baPWV burden. The absence of significant mediation by GFR in women implies that other mechanisms—potentially hormonal, endothelial, or structural differences in vascular aging—may be more dominant drivers linking blood pressure to arterial stiffening in this population.

During the mediation analysis, we used the Bootstrap method to ensure the robustness and reliability of the results. Our repeated sampling revealed a statistically significant mediation effect of renal function, indicating that both systolic and diastolic blood pressure influence atherosclerosis through renal function. These findings are consistent with previous research, which underscores the importance of considering renal function in assessing cardiovascular disease risk (23, 24).Consequently, monitoring and enhancing renal function should be prioritized in future clinical practice as an effective strategy to reduce the risk of atherosclerosis.

## 5 Conclusion

The purpose of this study was to explore the mediating relationship between blood pressure, renal function, and subclinical atherosclerosis in young and middle-aged non-hypertensive individuals. By analyzing blood pressure, the renal function, and cardiovascular health status of this population, we found that although hypertension was not diagnosed, blood pressure within the normal range—particularly at the higher end—was still significantly correlated with the risk of cardiovascular disease. Moreover, renal function played an important mediating role between blood pressure and subclinical atherosclerosis. This study sought to clarify how renal function may mediate the relationship between blood pressure and atherosclerosis, offering new insights for the early identification and intervention of cardiovascular diseases. The results show that glomerular filtration rate (GFR) is both a crucial measure of kidney function and a significant biomarker for atherosclerosis risk; specifically, elevated systolic and diastolic blood pressure may influence changes in brachial-ankle pulse wave velocity (baPWV) by affecting GFR. The reliance on GFR-mediated pathways in men could contribute to their generally higher baseline baPWV burden. These findings highlight the need to monitor and manage renal function in clinical practice to lower atherosclerosis risk.

The significance of this study lies in offering new perspectives on cardiovascular health in young and middle-aged, non-hypertensive individuals, particularly regarding the early identification and intervention of subclinical atherosclerosis. By examining the link between renal function and blood pressure, this study offers valuable theoretical insights and practical recommendations to guide future clinical research.

Of course, this study has certain limitations. The research subjects are mainly employed individuals from the Shanghai region, and this specific demographic distribution may limit the applicability of the research results to other populations with different demographic characteristics. Additionally, the cross-sectional design limits the comprehensive collection of all potential confounding variables affecting the relationship between exposure and outcome. Even with adjustments for some known confounding factors, there may still be unknown or hard-to-measure confounding factors that interfere with the analysis results. To enhance the generalizability of the research findings, future studies could focus on strengthening multi-center collaboration by uniting different regions and types of research institutions. They could also expand the geographical coverage of the sample and include research subjects with diverse socioeconomic backgrounds and cultural characteristics. These measures would effectively reduce the homogeneity of the sample.

To build on these findings, future research should investigate the relationship between renal function and atherosclerosis using larger sample sizes and longitudinal designs. Such studies will help validate the current results and explore potential interventions. Additionally, examining how renal function interacts with other cardiovascular risk factors will enhance our understanding of cardiovascular disease mechanisms, informing more precise clinical intervention strategies. Through further research, we aim to provide clear and effective guidance for cardiovascular health management in young and middle-aged individuals, ultimately reducing the incidence of cardiovascular disease.

## Data Availability

The original contributions presented in the study are included in the article/Supplementary Material, further inquiries can be directed to the corresponding author.
